# Catecholaminergic Gene Polymorphisms Are Associated with GI Symptoms and Morphological Brain Changes in Irritable Bowel Syndrome

**DOI:** 10.1371/journal.pone.0135910

**Published:** 2015-08-19

**Authors:** Alexa Orand, Arpana Gupta, Wendy Shih, Angela P. Presson, Christian Hammer, Beate Niesler, Nuwanthi Heendeniya, Emeran A. Mayer, Lin Chang

**Affiliations:** 1 Oppenheimer Center for the Neurobiology of Stress, David Geffen School of Medicine, University of California Los Angeles, Los Angeles, California, United States of America; 2 Department of Biostatistics, David Geffen School of Medicine, University of California Los Angeles, Los Angeles, California, United States of America; 3 Division of Epidemiology, Department of Internal Medicine, University of Utah, Salt Lake City, Utah, United States of America; 4 Institute of Human Genetics, Department of Human Molecular Genetics, University of Heidelberg, Heidelberg, Germany; University of Illinois-Urbana Champaign, UNITED STATES

## Abstract

**Background:**

Genetic and environmental factors contribute to the pathophysiology of irritable bowel syndrome (IBS). In particular, early adverse life events (EALs) and the catecholaminergic system have been implicated.

**Aims:**

To investigate whether catecholaminergic SNPs with or without interacting with EALs are associated with: 1) a diagnosis of IBS, 2) IBS symptoms and 3) morphological alterations in brain regions associated with somatosensory, viscerosensory, and interoceptive processes.

**Methods:**

In 277 IBS and 382 healthy control subjects (HCs), 11 SNPs in genes of the catecholaminergic signaling pathway were genotyped. A subset (121 IBS, 209 HCs) underwent structural brain imaging (magnetic resonance imaging [MRI]). Logistic and linear regressions evaluated each SNP separately and their interactions with EALs in predicting IBS and GI symptom severity, respectively. General linear models determined grey matter (GM) alterations from the SNPs and EALs that were predictive of IBS.

**Results:**

1) Diagnosis: There were no statistically significant associations between the SNPs and IBS status with or without the interaction with EAL after adjusting for multiple comparisons. 2) Symptoms: GI symptom severity was associated with *ADRA1D* rs1556832 (P = 0.010). 3) Brain morphometry: In IBS, the homozygous genotype of the major *ADRA1D* allele was associated with GM increases in somatosensory regions (FDR q = 0.022), left precentral gyrus (q = 0.045), and right hippocampus (q = 0.009). In individuals with increasing sexual abuse scores, the *ADRAβ2* SNP was associated with GM changes in the left posterior insula (q = 0.004) and left putamen volume (q = 0.029).

**Conclusion:**

In IBS, catecholaminergic SNPs are associated with symptom severity and morphological changes in brain regions concerned with sensory processing and modulation and affect regulation. Thus, certain adrenergic receptor genes may facilitate or worsen IBS symptoms.

## Introduction

Irritable bowel syndrome (IBS) affects approximately 10.5% of the population (6.6% of men and 14.0% of women).[1] As currently defined, IBS likely represents a heterogeneous group of disorders presenting with a similar symptom pattern, which is characterized by abdominal pain, altered bowels habits and gastrointestinal (GI) symptom-specific anxiety.[2] Several studies suggest an interaction between genetic and environmental factors (including early adverse life events [EALs]) in the development of altered brain-gut interactions in IBS.[3–6]

Single nucleotide polymorphisms (SNPs) residing in genes of catecholaminergic signaling systems (including genes encoding adrenergic receptors and catechol-o-methyltransferase [*COMT*]) have been implicated in the pathophysiology of chronic pain disorders.[7–9] *COMT* has broad biological functions, including the metabolism of both catecholamines (epinephrine, norepinephrine, and dopamine) and enkephalins.[10] Polymorphisms in the *COMT* gene can influence catecholamine and enkephalin levels, which have been associated with dysregulation of pain modulatory systems in visceral pain.[3,8] SNPs in adrenergic receptors have also been implicated in the pathophysiology of IBS and chronic pain.[7,11] In a study of five adrenergic receptor genes, genotype frequences of SNPs residing in the alpha adrenergic-β2 (*ADRAβ2*) and alpha adrenergic-1D (*ADRA1D*) receptor genes differed significantly between patients with interstitial cystitis, also known as painful bladder syndrome (which commonly coexists with IBS) and healthy controls.[9] *ADRAβ2* polymorphisms were found to predict temporomandibular disorder status in 386 healthy individuals.[7] These studies show that while catecholaminergic SNPs alone do not cause IBS or any other chronic pain disorders, they may play a role by interacting with other genes and environmental factors such as EALs.

Several recent reports have demonstrated neuroplastic grey and white matter differences between IBS patients and healthy control subjects.[12–15] For example, IBS patients showed both smaller and larger regional grey matter volumes (GMV)[12] and increased cortical thickness (CT)[13], in particular in regions and networks involved in sensory processing (somatosensory, precentral and postcentral gyrus; viscerosensory, posterior insula), and decreased CT in regions associated with affect regulation (e.g., hippocampus, subgenual anterior cingulate cortex [sgACC]). Other studies have shown that grey matter (GM) volume alterations in pain and interoceptive regions (insula subregions) correlated with visceral sensitivity[15] and GI symptom severity.[14] It is currently not known if the observed brain changes are a consequence of chronic pain, or if they are genetically/environmentally determined vulnerability factors that increase the likelihood of developing persistent symptoms in response to certain triggering events, such as gastroenteric infections or injuries.[14–16] Several molecular mechanisms (some related to chronic stress) including neuronal or glial cell loss, reduction in the density of dendritic spines, and neurogenesis have been proposed to explain some of the neuroplastic changes.[13–15]

Although studies have investigated the relationship between catecholaminergic SNPs and some chronic pain disorders, the interactions between these SNPs, EALs, and resultant changes in brain structures in IBS patients have not been reported. The catecholaminergic SNPs evaluated in this study were chosen based on an extensive review of the literature (Table 1). We searched specifically for SNPs associated with IBS and comorbid conditions in which studies suggest that autonomic nervous system dysregulation plays a role in the pathophysiology of the disease process. [8,17–19] Our aim was to investigate whether catecholaminergic SNPs with or without interacting with EALs are associated with: 1) a diagnosis of IBS, 2) IBS symptoms and 3) morphological alterations in brain regions associated with somatosensory, viscerosensory, and interoceptive processes.

**Table 1:** Catecholaminergic SNPs.

*Gene*	SNPs	Name	Function
*COMT*	rs4680 (Val158Met), rs6269, rs174697	Catechol-o-methyltransferase	Degrades catecholamines (Norepinephrine, Epinephrine, Dopamine)
*ADRAβ2*	rs1432622, rs2400707, rs1042717, rs1042713	Adrenergic β2 receptor	Mediates sympathetic activity
*ADRA1D*	rs1556832, rs946188	Adrenergic 1D receptor	Mediates sympathetic activity
*ANKK1*	rs1800497	DRD2/ANKK1- Taq1A	Mediates dopamine activity
*DRD3*	rs6280	D3 dopamine receptor	Mediates dopamine activity

## Materials and Methods

### Study Subjects and Recruitment

IBS patients and healthy controls (HCs) were primarily recruited from community advertisements although some patients were recruited from a functional bowel disorders clinic at a university hospital setting. Subjects were between 18–55 years of age and underwent a medical history and physical examination by gastroenterologist or nurse practitioner with expertise in IBS. The diagnosis of IBS and bowel habit subtyping was determined by Rome III criteria,[20] the absence of other chronic GI conditions, and by a clinician with expertise in IBS. Demographic information including age, gender, body mass index (BMI), and race and ethnicity of subjects was collected. Race and ethnicity was determined using the NIH policy guidelines for collecting and presenting data on race and ethnicity for all NIH research studies.[21] Exclusion criteria comprised pregnancy, substance abuse, abdominal surgery, tobacco dependence (smoked half a package of cigarettes or more daily), psychiatric illness, and extreme strenuous exercise (exercised one hour or more per day). HCs had no history of IBS, other chronic pain disorders, or psychiatric illnesses, and were not taking beta-adrenergic blockers or centrally acting drugs (antidepressants, anxiolytics, narcotics).

All subjects were compensated for participating in the study, and written informed consent was obtained from all subjects. The study was approved by the University of California Los Angeles (UCLA) Institutional Review Board and was conducted in accordance with the institutional guidelines regulating human subjects research.

### Symptom Measures

A bowel symptom questionnaire was used to assess the presence and severity of IBS symptoms (abdominal pain, bloating, usual and overall IBS symptom severity) and determine fulfillment of the Rome III diagnostic criteria.[22] Severity of abdominal pain, IBS symptoms, and sensation of bloating during the past week were assessed on a scale from 0 (no pain) to 20 (most intense pain imaginable).[23] Usual IBS symptom severity was ranked on a 5-point scale, which included: none, mild, moderate, severe or very severe symptoms.[23] EALs were measured using the Early Trauma Inventory- Short Form Self Report (ETI-SR).[24] The ETI-SR questionnaire assesses 27 individual items within 4 EAL domains: general trauma (11 questions), physical trauma (5 questions), emotional trauma (5 questions) and sexual abuse (6 questions). Items scored “yes” = 1 point and “no” = 0 points. The total score ranges from 0–27, and has been used in IBS to measure number of EAL events.[23]

Other on-GI clinical traits were assessed using the following scales: current anxiety and depression symptoms (Hospital Anxiety and Depression Scale [HAD]),[25] GI-symptom specific anxiety (Visceral Sensitivity Index [VSI]),[26] somatic symptoms (Patient Health Questionnaire [PHQ-15]), [27] quality of life- short form (SF-12-physical and mental), [28] trait anxiety, [29] bowel symptom questionnaire (BSQ), [30] and personality inventory (NEO scales). [31] In addition, all the subjects had previously undergone a structured psychiatric interview (MINI) to measure past or current psychiatric illness.[32,33]

### DNA Collection

DNA extracted from salivary samples of IBS patients and HCs was processed and assessed by the UCLA Biological Samples Processing Core using the Autopure LS Nucleic Acid Purification instrument (Gentra Systems, Inc., Minneapolis, MN.) The *COMT* polymorphism (rs174697) was genotyped using a 5′ nuclease assay to discriminate between the two alleles A/G (Taqman SNP Genotyping Assay C_2255328_10, Applied Biosystems Inc.). Polymerase chain reactions were performed using 5-μL reaction volumes in 384-well plates with 5 ng of DNA. The standard protocol provided with the kit was followed. End point reads of fluorescence levels were obtained with an ABI 7900HT Sequence Detection System. The SNPType genotyping was done on the other 10 SNPs using the Fluidigm Biomark system. First, 10–60 ng of DNA was pre amplified using Qiagen Multiple PCR master mix. This was then diluted and used for amplification as starting material. Next, the samples and assays were loaded onto GT 96*96 Dynamic array and processed per Fluidigm protocol. The assays used were designed using Fluidigm’s proprietary technology. The genotyping calls were made using Fluidigm SNP genotyping software. The SNPs rs4680 and rs6280 were genotyped with the KASP genotyping system (KBiosciences, Ltd, Hoddesdon, UK) as recommended by the manufacturer using customized assays as outlined previously.[34]

#### Brain imaging

Structural brain images were obtained from a subset of IBS patients and HCs in whom DNA samples were collected.

#### MRI structural data acquisition

High resolution structural images were acquired with a magnetization-prepared rapid acquisition gradient echo (MP-RAGE) sequence with the following parameters: repetition time (TR) = 2200ms, echo time (TE) = 3.26ms, slice thickness = 1mm, 176 slices, 256 x 256 voxel matrices, and 1.0×1.0×1.0mm voxel size.

#### Cortical Thickness (CT) and Grey Matter Volume (GMV)

The University of Southern California Laboratory of Neuroimaging pipeline (http://pipeline.loni.usc.edu/), a graphical workflow environment was utilized for image preprocessing and CT and GMV analyses. All structural scans were first converted from DICOM to NIFTI, a format that could be analyzed, including intensity inhomogeneity correction,[35] skull-stripping,[36,37] and cortical surface modeling.[38] Gray matter thickness and volume were estimated using a well-validated method[39] implemented in FreeSurfer 4.0[38] (available free to the public, http://surfer.nmr.mgh.harvard.edu/fswiki and http://ucla.in/xSQPqT). The obtained CT and GMV maps were registered to International Consortium for Brain Mapping (ICBM) brain surface and then vertex-wise correspondences were established between all cortical surface models using a conformal metric optimization method.[40] In order to increase statistical power, an 8 mm heat kernel[41] was applied to smooth the realigned thickness and volume maps. Total brain volume (TBV) values were also obtained from the same pipeline. Data is available on our Pain website (http://painrepository.org/) as part of the Pain and Interoception Network (PAIN) repository.

### Statistical Analysis

#### Genetic analysis

Eleven catecholaminergic SNPs residing in five genes were genotyped (Table 1). Hardy–Weinberg equilibrium and three different genetic models (additive, dominant, and recessive) were tested for each SNP. The dominant genetic model (one or more copies of the minor allele vs. no copies) was found to be the most statistically significant in unadjusted comparisons with IBS status, and thus this model was utilized for all SNPs to focus downstream analyses. Individual logistic regressions were used to predict the odds of IBS status from each SNP while controlling for race and sex. We also evaluated the effect of the interactions between EAL and each SNP in predicting IBS status. As an exploratory analysis, we also repeated this model controlling for HAD anxiety and depression scores. Any EAL x SNP interactions that were significant were further characterized by running separate logistic regression models within each genotype group (homozygous major and heterozygous/homozygous minor). Significance for the primary analysis was p<0.002 to adjust for analyzing ETI-SF (EAL) total score and the four subdomains and the five genes. We did not control for multiple comparisons in our secondary aim (the effect of the catecholaminergic SNPs on GI symptoms) because it was an exploratory analysis. The 0.05 significance level was used to define important interactions for the brain imaging analysis.

We also conducted exploratory analyses comparing an additional 26 clinical traits (which included GI symptoms and non-GI symptoms) with each SNP within the IBS group. Exploratory factor analysis (EFA) was used to mitigate multiple testing for this explorative analysis by reducing the dimensionality of the 26 clinical traits down to a few representative latent factors. A scree plot was used to estimate the number of factors. The factors were then extracted using principal-component analysis, and we determined the optimal configuration of items on factors using a varimax rotation. Finally, we tested each of the latent factors with each SNP in linear regression models that controlled for race and sex. Significance was defined by p<0.05. All statistical analyses were performed using R version 3.0.2 (http://cran.r-project.org/) and all tests were two-tailed.

#### Brain imaging analysis

To test the specific hypotheses, linear contrast analysis within the framework of the general linear models (GLM) were constructed to examine the main and interactive effects of genetic variation (in the SNPs found to predict IBS and correlate with GI symptoms) with EALs on the CT and GMV of specific brain regions, controlling for race, sex and total brain volume (TBV). Linear contrast analyses specified to test how IBS compared to HCs. A GLM was chosen for these analyses in order to describe a large number of responses (morphological measures) as a function of first early adverse life events and then GI symptoms. An exploratory analysis covariate analysis with depression and anxiety scores was conducted in the GLM model specified above, in order to determine if these mood measurements could be confounding factors. Correlations were also conducted between GM alterations and IBS specific symptoms. Twenty specific brain regions selected were based on previously published papers where significant differences between IBS and HCs were found (a summary of these brain regions and their functions has been provided in (Table 2 **).**[12,13] Corrections for multiple brain regions of interest (ROI) comparisons were made using the false discovery rate (FDR), where a FDR q <0.05 was considered significant.[42,43]

**Table 2:** Summary of brain regions used in the analyses and their associated functions.

Brain Regions	Associated Function
Insula (anterior insula [aINS] and mid insula [mINS])	Interoceptive integration, and viscerosensory processing
Posterior Insula (pINS)	Primary viscerosensory cortex
Anterior Cingulate Cortex [ACC] (subgenual [sgACC], pregenual [pgACC])	Affect regulation
Anterior Mid Cingulate Cortex (aMCC)	Endogenous pain modulation
Postcentral Gyrus (PostCG)	Sensory processing, integration and modulation
Precentral Gyrus (PreCG)	Motor control
Amygdala (Amyg)	Affect regulation, autonomic output, endogenous pain modulation
Hippocampus (Hipp)	Affect control, memory
Putamen	Sensory processing, integration and modulation, reward, and learning
Superior Frontal Gyrus (SFG)	Cognitive modulation
Gyrus Rectus	Cognitive modulation
Middle Orbital Gyrus (mOFG)	Cognitive modulation

## Results

### Baseline Clinical Characteristics

There were 659 subjects who participated in the study. This group was comprised of 277 IBS patients (mean age 36.4 yrs, 75.2% women) and 382 HCs (mean age 30.3 yrs, 73.2% women). Of these 659 subjects, a subset of 330 right-handed individuals (121 [43.7%] IBS, 209 [54.7%] HCs) completed structural MRI scans. The baseline clinical characteristics of the larger group and subset who underwent brain imaging are summarized in Table 3. In both the larger dataset and brain imaging subset, the IBS patients had significantly higher scores on the Hospital Anxiety and Depression Scale (HAD), ETI-SR (EALs) and visceral sensitivity index (VSI) than HCs. In the larger dataset, the IBS patients also had a significantly higher mean age and ETI-SR sexual abuse score. Except for one IBS subject on chronic low dose amitriptyline (50 mg daily), no other patients were taking centrally acting medications.

**Table 3:** Clinical Characteristics of Subjects.

	Genetics Dataset			Brain Imaging Dataset		
	HCs (N = 381)	IBS (N = 278)		HCs (N = 205)	IBS (N = 115)	
Variable	Mean (SD)	Mean (SD)	p-value	Mean (SD)	Mean (SD)	p-value
Age (yrs.)	30.29 (10.68)	36.4 (12.38)	<0.001	30.95 (11.12)	31.92 (10.2)	0.178
BMI (SD)	24.55 (4.7)	25.17 (5.01)	0.101	24.28 (4.31)	23.71 (3.99)	0.346
Female: N (%)	279 (73.22%)	209 (75.18%)	0.59	165 (80.49%)	85 (73.91%)	0.221
Race			<0.001			0.002
Caucasian	154 (41.4%)	166 (61.5%)		82 (40.8%)	68 (60.2%)	
Asian	106 (28.4%)	32 (11.9%)		66 (32.9%)	20(17.7%)	
African American	56 (15.1%)	37 (13.7%)		29 (14.4%)	9 (8.0%)	
Other/Mixed	56 (15.1%)	35 (12.9%)		24 (11.9%)	16 (14.1%)	
HAD Anxiety (0–21)	3.35 (2.77)	7.35 (4.28)	<0.001	3.18 (2.7)	6.49 (3.59)	<0.001
HAD Depression (0–21)	1.32 (1.75)	3.83 (3.39)	<0.001	1.36 (1.78)	3.00 (2.98)	<0.001
VSI Score (0–90)	2.51 (5.07)	34.75 (16.35)	<0.001	2.81 (5.24)	33.37 (14.76)	<0.001
ETI General Trauma Score (0–11)	1.51 (1.67)	2.47 (2.18)	<0.001	1.47 (1.52)	2.06 (1.77)	0.002
ETI Physical Score (0–5)	1.14 (1.43)	1.58 (1.60)	<0.001	1.03 (1.34)	1.33 (1.45)	0.058
ETI Emotional Score (0–5)	0.71 (1.33)	1.58 (1.77)	<0.001	0.68 (1.28)	1.39 (1.68)	<0.001
ETI Sexual Score (0–6)	0.36 (0.96)	0.76 (1.53)	<0.001	0.37 (0.97)	0.49 (1.14)	0.245
IBS Bowel Habit Subtype: N (%)						
Constipation		62 (22.30%)			31 (26.96%)	
Diarrhea		65 (23.38%)			24 (20.87%)	
Mixed		138 (49.64%)			55 (47.83%)	
Unspecified		13 (4.68%)			5 (4.35%)	
BSQ Symptoms (SD)						
Overall Severity (0–20)		10.17 (4.56)			10.67 (4.34)	
Abdominal Pain (0–20)		9.34 (5.12)			9.77 (5.04)	
Bloating (0–20)		11.00 (5.36)			12 (5.15)	
Usual Severity of IBS (1–5)		3.13 (0.81)			3.11 (0.78)	
Age of Onset of IBS (yrs)		20.15 (12.04)			19.39 (9.95)	
Severity Duration (yrs.)		11.68 (11.2)			9.96 (8.5)	

Abbreviations: HCs = healthy controls, IBS = irritable bowel syndrome, BMI = body mass index, HAD = hospital anxiety & depression, ETI = early trauma inventory VSI = visceral sensitivity index Add all of the other clinical traits

### Association of Catecholaminergic SNPs and IBS Diagnosis

**Fig 1:**
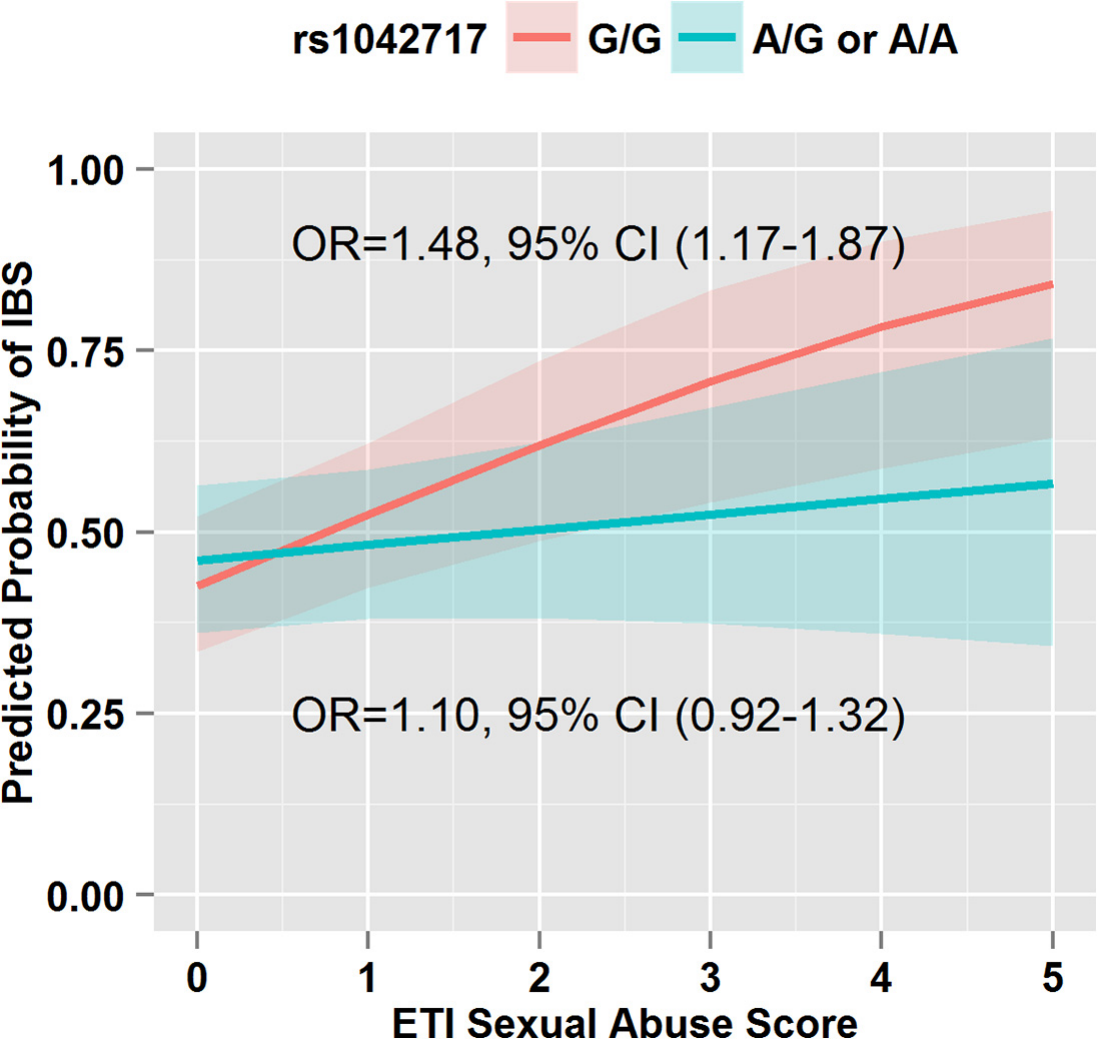
The Presence of the Minor Allele in the *ADRAβ2* rs1042717 Gene When Interacted with Increasing Sexual Scores is Protective Against an IBS Diagnosis. *ADRAβ2* rs1042717 (A/G or A/A) in the presence of an elevated ETI-SR sexual abuse score results in a lower likelihood of IBS (interaction term OR = 0.73, 95% CI: 0.55–0.98, p = 0.039). Odds ratios and 95% CIs for a unit increase in ETI-SR sexual abuse score are reported from separate logistic regression models run within each genetic subgroup.

**Fig 2:**
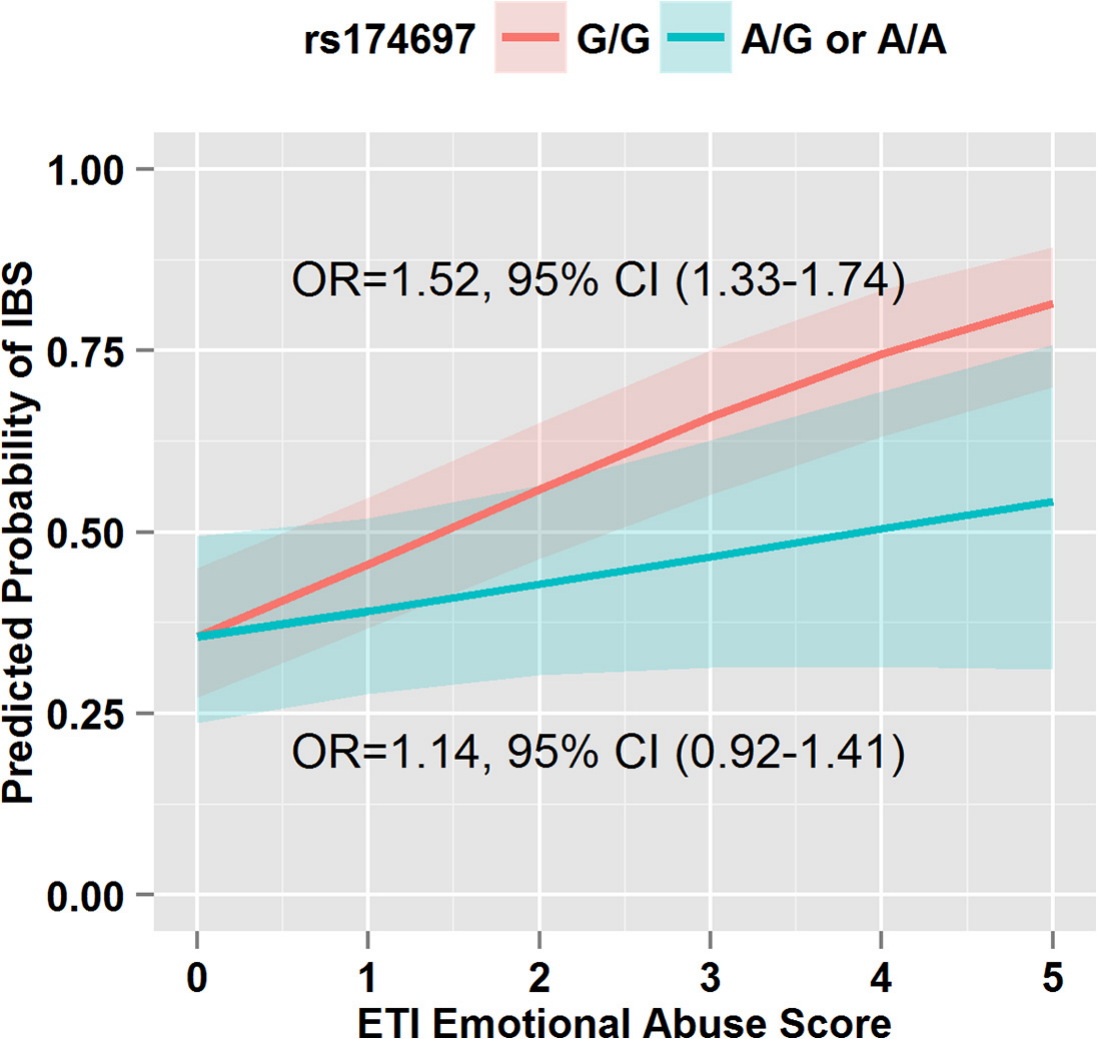
The Presence of the Minor Allele in the *COMT* rs174697 Gene When Interacted with Increasing Emotional Scores is Protective Against an IBS Diagnosis. *COMT* SNP rs174697 (A/G or A/A) in the presence of an elevated ETI-SR emotional abuse score results in a lower likelihood of IBS than the G/G (major allele) genotype (interaction term OR = 0.76, 95% CI: 0.60–0.99, p = 0.041). Odds ratios and 95% CIs for a unit increase in ETI-SR emotional abuse score are reported from separate logistic regression models run within each genetic subgroup.

There was no significant difference in genotype frequencies between IBS and HCs for the 11 SNPs in the overall group (Table 4) and in the subset with brain imaging (S1 Table). Furthermore, there were no significant SNP and EAL interactions after correcting for multiple comparisons. However, there were two SNP and EAL interaction that had uncorrected p-values <0.05 and were assessed in the brain imaging analysis (see below). Individuals who were homozygous carriers of the *ADRAβ2* rs1042717 major G allele were associated with increased likelihood of having IBS with increasing sexual abuse score as compared to individuals who carried the minor A allele in a heterozygous or homozygous manner (odds ratios [OR]: 1.48 [95% CI 1.17–1.87] vs. 1.10 [0.92–1.32], p = 0.039) (Fig 1). Similarly, compared to the individuals who presented with a heterozygous or homozygous state of the minor A allele (i.e., had at least one minor A allele) for *COMT* rs174697, individuals who carried the homozygous major G allele (i.e., had both copies of the major G allele) had a higher likelihood of having IBS if they had an increasing emotional abuse score (OR: 1.52 [1.33–1.74] vs. 1.14 [0.92–1.42], p = 0.041) (Fig 2). After controlling for HAD anxiety and depression scores, the interactions between EAL scores and *ADRAβ2* rs1042717 and *COMT* rs174697 were not statistically significant.

**Table 4:** Prevalence of Catecholaminergic Genotypes in IBS Patients and Healthy Controls.

SNP (Gene)	Healthy Controls				IBS Patients			
rs1556832 (*ADRA1D)*	**C/C**	**C/T**	**T/T**	**No Call**	**C/C**	**C/T**	**T/T**	**No Call**
	166 (43.6%)	159 (41.7%)	51 (13.4%)	5 (1.3%)	93 (33.5%)	134 (48.2%)	47 (16.9%)	4 (1.4%)
rs946188 (*ADRA1D)*	**A/A**	**A/G**	**G/G**	**No Call**	**A/A**	**A/G**	**G/G**	**No Call**
	191 (50.1%)	162 (42.5%)	20 (5.2%)	8 (2.1%)	140 (50.4%)	118 (42.4%)	11 (4%)	9 (3.2%)
rs1432622 (*ADRAβ2)*	**C/C**	**C/T**	**T/T**	**No Call**	**C/C**	**C/T**	**T/T**	**No Call**
	164 (43%)	161 (42.3%)	50 (13.1%)	6 (1.6%)	106 (38.1%)	128 (46%)	40 (14.4%)	4 (1.4%)
rs2400707 (*ADRAβ2)*	**G/G**	**A/G**	**A/A**	**No Call**	**G/G**	**A/G**	**A/A**	**No Call**
	164 (43%)	161 (42.3%)	51 (13.4%)	5 (1.3%)	106 (38.1%)	129 (46.4%)	41 (14.7%)	2 (0.7%)
rs1042717 (*ADRAβ2)*	**G/G**	**A/G**	**A/A**	**No Call**	**G/G**	**A/G**	**A/A**	**No Call**
	164 (43%)	161 (42.3%)	51 (13.4%)	5 (1.3%)	106 (38.1%)	129 (46.4%)	41 (14.7%)	2 (0.7%)
rs1042713 (*ADRAβ2)*	**G/G**	**A/G**	**A/A**	**No Call**	**G/G**	**A/G**	**A/A**	**No Call**
	120 (31.5%)	165 (43.3%)	84 (22%)	12 (3.1%)	95 (34.2%)	123 (44.2%)	47 (16.9%)	13 (4.7%)
rs1800497 (*ANKK1)*	**C/C**	**C/T**	**T/T**	**No Call**	**C/C**	**C/T**	**T/T**	**No Call**
	192 (50.4%)	148 (38.8%)	36 (9.4%)	5 (1.3%)	159 (57.2%)	93 (33.5%)	24 (8.6%)	2 (0.7%)
rs6269 (*COMT)*	**A/A**	**A/G**	**G/G**	**No Call**	**A/A**	**A/G**	**G/G**	**No Call**
	171 (44.9%)	158 (41.5%)	46 (12.1%)	6 (1.6%)	107 (38.5%)	129 (46.4%)	38 (13.7%)	4 (1.4%)
rs4680 (*COMT)*	**G/G**	**A/G**	**A/A**	**No Call**	**G/G**	**A/G**	**A/A**	**No Call**
	128 (33.6%)	181 (47.5%)	68 (17.8%)	4 (1%)	98 (35.3%)	111 (39.9%)	59 (21.2%)	10 (3.6%)
rs174697 (*COMT)*	**G/G**	**A/G**	**A/A**	**No Call**	**G/G**	**A/G**	**A/A**	**No Call**
	250 (65.6%)	107 (28.1%)	19 (5%)	5 (1.3%)	219 (78.8%)	52 (18.7%)	6 (2.2%)	1 (0.4%)
rs6280 (*DRD3)*	**T/T**	**C/T**	**C/C**	**No Call**	**T/T**	**C/T**	**C/C**	**No Call**
	142 (37.3%)	169 (44.4%)	66 (17.3%)	4 (1%)	107 (38.5%)	123 (44.2%)	44 (15.8%)	4 (1.4%)

### Association of Catecholaminergic SNPs and Symptoms

Five latent factors were identified using factor analysis for the 26 clinical traits within the IBS population. The latent factors were summarized as psychological traits, somatic symptoms, symptom severity, EALs, and personality traits. Within the IBS patient group, those who were homozygous for the major allele of the *ADRA1D* SNP rs1556832 had higher IBS symptom severity (i.e. overall severity, abdominal pain, bloating, and usual severity) compared to those who were heterozygous or homozygous for the minor allele after adjusting for race and sex (p = 0.010).

### Association of Catecholaminergic SNPs with Regional Brain Structural Changes

**Fig 3:**
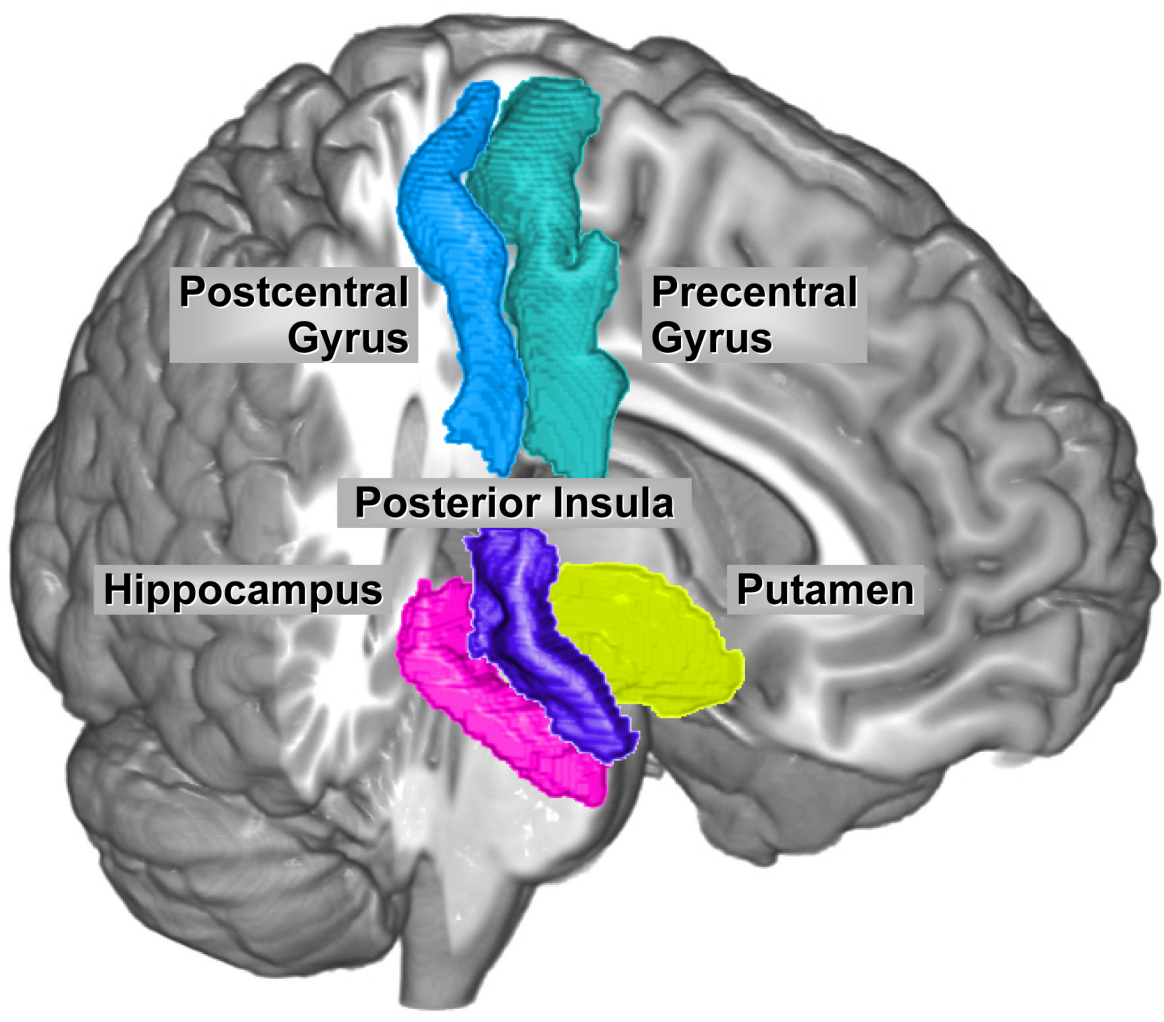
Brain Regions with Significant Associations with Catecholaminergic SNPs. The increase in cortical thickness of left posterior insula was demonstrated for the homozygous major allele *ADRAβ2* rs1042717 genotype in the presence of increasing sexual abuse scores (q = 0.004). The decrease in the volume of the left putamen was demonstrated for the homozygous and heterozygous *ADRAβ2* rs1042717 minor allele genotypes in the presence of increasing sexual abuse scores (q = 0.029). An increase in the cortical thickness of the left postcentral gyrus was demonstrated in the presence of increasing emotional abuse scores, regardless of disease status or *COMT* SNP rs174697 genotype (q = 0.042). Increases in the volumes of left postcentral gyrus (q = 0.022), left precentral gyrus (q = 0.045), and right hippocampus (q = 0.009) were demonstrated for the homozygous *ADRA1D* SNP rs1556832 major allele genotype in IBS patients. The association of *ADRA1D* SNP rs1556832 with the right hippocampus volume was not significant after controlling for HAD depression scores.

Based on our above findings, we determined brain structural changes correlated with: 1) *ADRAβ2* SNP rs1042717 with and without taking sexual abuse score into account, 2) *COMT* SNP rs174697 with and without taking emotional abuse score into account, and 3) *ADRA1D* SNP rs1556832 in IBS. All brain regions with significant associations with catecholaminergic SNPs are shown in Fig 3 The homozygous major allele *ADRAβ2* rs1042717 genotype was associated with decreases in the CT of the primary interoceptive cortex (left posterior insula) in the presence of increasing sexual abuse scores (q = 0.004). On the other hand, the homozygous and heterozygous *ADRAβ2* rs1042717 minor allele genotypes were associated with decreases in left putamen volume in the presence of increasing sexual abuse scores (q = 0.029). Regardless of disease status or *COMT* SNP rs174697 genotype, increasing emotional abuse scores were associated with increases in the CT of the left postcentral gyrus (primary somatosensory cortex) (q = 0.042). In IBS patients only, the homozygous *ADRA1D* SNP rs1556832 major allele genotype was associated with increases in the volume of the somatosensory regions (left postcentral [q = 0.022], the left precentral gyrus [q = 0.045]), and the right hippocampus (an affective region [q = 0.009]).

To determine the potential influence of anxiety and depression symptoms on the observed results, additional covariate analyses were performed. When controlling for depression and anxiety scores, all differences were maintained for the *ADRAB2* rs1042717 SNP on the cortical thickness for the left posterior insula and the volume of the left putamen. When controlling for depression and anxiety scores, the differences for the *COMT* SNP rs174697 on the cortical thickness for the left postcentral gyrus remained significant. However, in IBS patients only, when controlling for depression score the difference for the *ADRA1D* SNP rs1556832 on the volume of the left hippocampus was no longer significant. When controlling for anxiety score in IBS patients only, the difference for the *ADRA1D* SNP rs1556832 on all the initial findings remained significant.

### Correlation of Morphological Measures with GI Symptoms

Cortical thickness of the left somatosensory cortex (postcentral gyrus) was positively correlated with usual severity of IBS symptoms (r = 0.258, q = 0.023). Although there were other cortical thickness and grey matter volume measures in brain regions other than the somatosensory cortex (postcentral gyrus) that were significantly correlated with GI symptoms, they did not survived corrections for multiple comparisons.

## Discussion

We aimed to test the hypothesis that genetic and environmental factors interact to influence IBS status, GI symptomology and brain structure in a large, well phenotyped cohort of 659 IBS and HC subjects. This study showed that catecholaminergic SNPs were associated with IBS symptom severity and grey matter alterations in specific brain regions involved in sensory processing and affect regulation (Table 5). Despite the fact that altered catecholaminergic signaling is commonly associated with many chronic pain conditions and that EALs are common environmental factors associated with IBS, to our knowledge, this is the first demonstration of interactions of these factors in influencing symptom related morphological brain changes in a large cohort of IBS patients.

**Table 5:** Summary of Main Findings.

Gene	SNP	Association with IBS +/- EALs	Association with Symptoms	Associated Brain Regions
*COMT*	rs4680 (Val158Met)	-	-	-
	rs6269	-	-	-
	rs174697	HM with increasing emotional abuse score increased likelihood of IBS (p = 0.041 uncorrected)	-	HM, Hm &He = increased CT of left postcentral gyrus (All with increasing emotional abuse scores)
*ADRAβ2*	rs1432622	-	-	-
	rs2400707	-	-	-
	rs1042717	HM with increasing sexual abuse score increased likelihood of IBS (p = 0.039 uncorrected)	-	HM = Decreased CT of left posterior insula, Hm & He = Decreased left putamen volume (All with increasing sexual abuse scores)
	rs1042713	-	-	-
*ADRA1D*	rs1556832	-	HM individuals had higher IBS symptom severity (p = 0.010)	HM = increased volume of left postcentral gyrus, left precentral gyrus, and the right hippocampus in IBS patients
	rs946188	-	-	-
*ANKK1*	rs1800497	-	-	-
*DRD3*	rs6280	-	-	-

HM = homozygous major, Hm = homozygous minor, He = heterozygous, CT = cortical thickness

### Effect of Catecholaminergic SNPs on IBS Diagnosis and Symptoms

We did not find any statistically significant relationships between the catecholaminergic SNPs and the presence of IBS with or without the interaction with EALs after adjusting for multiple comparisons. However, the α-adrenergic SNP, *ADRA1D* rs1556832, was significantly associated with GI symptom severity. There is minimal data that describes the role of *ADRA1D* receptor activity in the GI tract. However, a previous study demonstrated that the genotype frequencies of *ADRA1D* and *ADRAβ2* SNP were associated with bladder pain syndrome/interstitial cystitis (BPS/IC), a chronic visceral pain disorder which often coexists with IBS[9] and which shows similar brain changes as IBS (unpublished observations). In addition, *ADRA1D* tissue expression was increased in patients with bladder outlet obstruction and in patients with lower urinary symptoms who had increased bladder sensitivity. [44] Thus, this receptor is thought to play a role in sensory afferent processing, and in contractile activity.[44] Further studies are needed to determine if *ADRA1D* receptors have a similar effect on colonic function and if they have a pathophysiologic role in IBS.

### Effect of EALs on Brain Structure

We found that regardless of disease status or *COMT* SNP rs174697 genotype, increasing emotional abuse scores were associated with increases in the CT of the primary somatosensory cortex.[45] Increased thickness of somatosensory cortex has been reported in several chronic pain conditions, including IBS, BPS/IC, TMD, migraine, and chronic back pain.[13,46–48] The mechanisms underlying this increase are incompletely understood but may include increased regional growth factor release in response to continuous sensory input.[46,49–51] Alternatively, one study showed that neuroplastic changes in the somatosensory cortex were present in HCs who had increased sensitivity to experimental pain stimuli, in the absence of pain disorders. Furthermore, sensitivity to acute pain and heat pain stimuli was positively correlated with the thickness of the somatosensory cortex,[52,53] suggesting that this cortical alteration may be a risk factor for the development of chronic pain disorders.

A positive history of EALs is associated with an increased likelihood of developing IBS and other chronic pain conditions,[54] as well as, increased pain perception and sensitivity.[55] In addition, GM alterations have been reported in adults with a history of EALs, including larger volumes in the amygdala and reduced volumes in the prefrontal and anterior cingulate cortices, hippocampus, and cerebellum.[56–59] Even though alternative explanations are possible, these structural findings may reflect the compromised corticolimbic inhibition thought to result from early trauma.[3] Consistent with these findings, we recently reported EAL-associated changes in the connectivity of a saliency network in the brain that included viscero- and somatosensory regions.[60] Furthermore, as both a history of EALs and cortical somatosensory increases have been observed in IBS previously, the observed correlation between EAL scores and cortical thickness found in this study provides a possible mechanistic insight into these brain changes.

### Interaction of Catecholaminergic SNPs, EALs and Diagnosis on Brain Structure

Individuals carrying the *ADRAβ2* SNP rs1042717 major allele in a homozygous state had a higher likelihood of having IBS if they had a higher early life sexual abuse score, compared to those with a dominant genotype (i.e., at least one copy of the minor allele). Although this was not statistically significant after adjusting for multiple comparisons, this is consistent with our finding that the dominant genotype has protective effects against decreases in both the volume of the left putamen (bidirectional connection with cortical control regions) and CT of the primary interoceptive cortex (pINS), with increasing sexual abuse scores in IBS patients.

Interestingly, repeated stress in rodents has been shown to enhance peripheral visceral nociceptive signaling via adrenergic receptors.[61–63] For example, elevated circulating epinephrine (acting on β2 adrenergic receptors) can signal directly to colonic dorsal root ganglion neurons, resulting in visceral hyperexcitability and hyperalgesia to repeated stress.[61] In another study, three haplotypes of the *ADRAβ2* receptor gene, which accounted for 98% of the study population (n = 386), were found to predict hyper- or hypo-functioning states of the *ADRAβ2* receptor, both of which resulted in an increased incidence of temporomandibular joint disorder.[7] This study suggests that genetic variability may predispose individuals to chronic pain disorders, a concept consistent with the current findings.

The homozygous *ADRA1D* SNP rs1556832 major allele genotype was associated with increases in CT of somatosensory regions in IBS patients. Since this SNP was the only one that correlated with GI symptom severity, it suggests that these brain changes in IBS may be involved in viscerosensory processing or pain modulation.

Similar to the current findings, previous studies have shown that compared to HCs, IBS patients have decreased volume of the putamen and decreased CT of the pINS,[12,13] and these findings are similar to those found in other chronic pain conditions.[13,46–48,64] Studies have also shown that in the presence of a history of EALs, neuroplastic decreases in other brain regions are evident.[65] When viewed together with the relationship between somatosensory cortex and EALs, the current findings suggest that the risk or resilience towards the negative effects of EALs on some regional grey matter are determined by the interaction of early environmental factors with genes and disease status.[66]

Preclinical studies have shown that alterations in the adrenergic and noradrenergic systems play key roles in mediating physiological and behavioral responses in the brain in response to EALs.[67] Perinatal stress, an established rodent model for EALs has been shown to result in increased norepinephrine output from the locus coeruleus,[68,69] and increases in plasma norepinephrine levels have been reported in IBS patients both at rest and under stressful conditions.[69] Increased noradrenergic modulations of brain regions that are associated with the regulation of emotion, fear, and anxiety have been reported.[70] One can speculate that the observed interactions between catecholaminergic SNPs and EALs may determine the degree of abnormal emotional arousal and related engagement of descending pain facilitatory pathways. Predominant descending facilitation of spinal sensory transmission may in turn result in a chronically enhanced ascending sensory input to sensory brain regions as previously suggested.[12,13,52] Such a mechanism of chronically enhanced visceral hypersensitivity would be consistent with the observed neuroplastic changes, the persistent hypervigilance towards GI signals,[3,69] and potentially explain the increased severity of GI symptoms seen in our study.[3]

### Conclusions and Possible Clinical Implications

In IBS patients, catecholaminergic SNPs were associated with IBS symptom severity and structural changes in brain regions that are involved in sensory processing and affect regulation, which have previously been described in IBS patients and in other comorbid pain conditions, such as PBS/IC. If replicated in other samples, some of the observed regional grey matter changes may represent endophenotypes, which are influenced by genetic and epigenetic factors and contribute to the complex clinical phenotype. In that case, these endophenotypes should be present in asymptomatic relatives of IBS patients, and may represent a vulnerability factor, which increases the risk of developing IBS under physical (infections, inflammation) or psychological stressors. The association of catecholaminergic SNPs with IBS with or without EALs did not meet statistical significance after accounting for multiple comparisons, and therefore further studies in larger samples are needed. In addition, future studies in other functional pain populations will be needed to determine if such endophenotypes are disease specific or represent a general risk factor, which predicts the development of chronic pain conditions.

## Supporting Information

S1 Table Prevalence of Catecholaminergic Genotypes in IBS Patients and Healthy Controls within subset of individuals with brain imaging. (DOCX)
