# Supplementary material for: Catecholaminergic Gene Polymorphisms Are Associated with GI Symptoms and Morphological Brain Changes in Irritable Bowel Syndrome
Source: PLoS One. 2015 Aug 19;10(8):e0135910. doi: 10.1371/journal.pone.0135910 (PMC4546052; doi:10.1371/journal.pone.0135910)
Supplement: S1 Table — (DOCX) [file pone.0135910.s001.docx]

**S1 Table. Prevalence of Catecholaminergic Genotypes in IBS Patients and Healthy Controls within subset of individuals with brain imaging.**

| **SNP** | **Gene** | **Healthy Controls** | | | | **IBS Patients** | | | |
| --- | --- | --- | --- | --- | --- | --- | --- | --- | --- |
| **rs1556832** | ***ADRA1D*** | **C/C** | **C/T** | **T/T** | **No Call** | **C/C** | **C/T** | **T/T** | **No Call** |
|  |  | 85 (41.5%) | 93 (45.4%) | 24 (11.7%) | 3 (1.5%) | 43 (37.4%) | 52 (45.2%) | 20 (17.4%) | 0 (0%) |
| **rs946188** | ***ADRA1D*** | **A/A** | **A/G** | **G/G** | **No Call** | **A/A** | **A/G** | **G/G** | **No Call** |
|  |  | 118 (57.6%) | 74 (36.1%) | 10 (4.9%) | 3 (1.5%) | 66 (57.4%) | 44 (38.3%) | 4 (3.5%) | 1 (0.9%) |
| **rs1432622** | ***ADRAB2*** | **C/C** | **C/T** | **T/T** | **No Call** | **C/C** | **C/T** | **T/T** | **No Call** |
|  |  | 91 (44.4%) | 85 (41.5%) | 27 (13.2%) | 2 (1%) | 48 (41.7%) | 57 (49.6%) | 10 (8.7%) | 0 (0%) |
| **rs2400707** | ***ADRAB2*** | **G/G** | **A/G** | **A/A** | **No Call** | **G/G** | **A/G** | **A/A** | **No Call** |
|  |  | 91 (44.4%) | 85 (41.5%) | 27 (13.2%) | 2 (1%) | 48 (41.7%) | 57 (49.6%) | 10 (8.7%) | 0 (0%) |
| **rs1042717** | ***ADRAB2*** | **G/G** | **A/G** | **A/A** | **No Call** | **G/G** | **A/G** | **A/A** | **No Call** |
|  |  | 91 (44.4%) | 85 (41.5%) | 27 (13.2%) | 2 (1%) | 48 (41.7%) | 57 (49.6%) | 10 (8.7%) | 0 (0%) |
| **rs1042713** | ***ADRB2*** | **G/G** | **A/G** | **A/A** | **No Call** | **G/G** | **A/G** | **A/A** | **No Call** |
|  |  | 63 (30.7%) | 84 (41%) | 52 (25.4%) | 6 (2.9%) | 37 (32.2%) | 53 (46.1%) | 20 (17.4%) | 5 (4.3%) |
| **rs1800497** | ***ANKK1*** | **C/C** | **C/T** | **T/T** | **No Call** | **C/C** | **C/T** | **T/T** | **No Call** |
|  |  | 97 (47.3%) | 87 (42.4%) | 18 (8.8%) | 3 (1.5%) | 72 (62.6%) | 34 (29.6%) | 9 (7.8%) | 0 (0%) |
| **rs6269** | ***COMT*** | **A/A** | **A/G** | **G/G** | **No Call** | **A/A** | **A/G** | **G/G** | **No Call** |
|  |  | 97 (47.3%) | 85 (41.5%) | 21 (10.2%) | 2 (1%) | 40 (34.8%) | 57 (49.6%) | 18 (15.7%) | 0 (0%) |
| **rs4680** | ***COMT*** | **G/G** | **A/G** | **A/A** | **No Call** | **G/G** | **A/G** | **A/A** | **No Call** |
|  |  | 64 (31.2%) | 99 (48.3%) | 40 (19.5%) | 2 (1%) | 40 (34.8%) | 50 (43.5%) | 23 (20%) | 2 (1.7%) |
| **rs6280** | ***DRD3*** | **T/T** | **C/T** | **C/C** | **No Call** | **T/T** | **C/T** | **C/C** | **No Call** |
|  |  | 76 (37.1%) | 86 (42%) | 41 (20%) | 2 (1%) | 50 (43.5%) | 50 (43.5%) | 15 (13%) | 0 (0%) |
| **rs174697** | ***COMT*** | **G/G** | **A/G** | **A/A** | **No Call** | **G/G** | **A/G** | **A/A** | **No Call** |
|  |  | 130 (63.4%) | 61 (29.8%) | 10 (4.9%) | 4 (2%) | 91 (79.1%) | 22 (19.1%) | 2 (1.7%) | 0 (0%) |
